# Synthesis of Zeolites Na-P1 from South African Coal Fly Ash: Effect of Impeller Design and Agitation

**DOI:** 10.3390/ma6052074

**Published:** 2013-05-16

**Authors:** Dakalo Mainganye, Tunde Victor Ojumu, Leslie Petrik

**Affiliations:** 1 Department of Chemical Engineering, Cape Peninsula University of Technology, P.O Box 652, Cape Town 8000, South Africa; E-Mail: dakalo.mainganye4@gmail.com; 2 Environmental and Nano Sciences Research Group, Department of Chemistry, University of the Western Cape, Private Bag X17, Bellville 7535, South Africa; E-Mail: lpetrik@uwc.ac.za

**Keywords:** fly ash, zeolites, impeller design, agitation, zeolites Na-P1

## Abstract

South African fly ash has been shown to be a useful feedstock for the synthesis of some zeolites. The present study focuses on the effect of impeller design and agitation rates on the synthesis of zeolite Na-P1 which are critical to the commercialization of this product. The effects of three impeller designs (4-flat blade, Anchor and Archimedes screw impellers) and three agitation speeds (150, 200 and 300 rpm) were investigated using a modified previously reported synthesis conditions; 48 hours of ageing at 47 °C and static hydrothermal treatment at 140 °C for 48 hours. The experimental results demonstrated that the phase purity of zeolite Na-P1 was strongly affected by the agitation rate and the type of impeller used during the ageing step of the synthesis process. Although zeolite Na-P1 was synthesized with a space time yield (STY) of 15 ± 0.4 kg d^−1^m^−3^ and a product yield of 0.98±0.05 g zeolites/g fly ash for each impeller at different agitation speeds, zeolite formation was assessed to be fairly unsuccessful in some cases due the occurrence of undissolved mullite and/or the formation of impurities such as hydroxysodalite with the zeolitic product. This study also showed that a high crystalline zeolite Na-P1 can be synthesized from South African coal fly ash using a 4-flat blade impeller at an agitation rate of 200 rpm during the ageing step at 47 °C for 48 hours followed by static hydrothermal treatment at 140 °C for 48 hours.

## 1. Introduction

Coal is the most abundant source of energy worldwide. South Africa is one of the countries that depend largely on coal as the main source of energy. About 90% of the electricity generated in South Africa is from coal-fired power stations. Combustion of coal produces residues known as coal combustion by-products (CCPs) of which fly ash is one of its principal components. Fly ash is a fine-grained inorganic powder residue; it is composed of spherical glassy particles which are derived from minerals formed during coal combustion. An estimated amount of 500 million tons of coal fly ash are produced worldwide annually due to power generation [1], of which South Africa contributed approximately 36.22 million tons to this amount in 2011 [2]. This value is expected to increase due to the increasing need for the generation of sufficient energy for the ever-increasing population and industrial energy requirements.

From the 36.22 million tons of waste ash that was generated in South Africa in 2011, only 5.5% was reused [2].The remaining unused ash presents a huge environmental problem; it is disposed of in ash dams and dumps, both of which can be regarded as unsightly, environmentally undesirable and/or a non-productive use of land resources, as well as posing an on-going financial burden through the need for their long-term maintenance. Fly ash has found limited application in South Africa; it is used as an additive in the production of cement and concrete and in land reclamation and restoration [1,3,4]. Therefore, current research efforts are directed towards exploring alternative uses of fly ash either to reduce the cost of disposal or to minimize the impact on the environment. Petrik and co-worker have shown that the co-disposal of fly ash and acid mine drainage can be used to neutralize the acidity of AMD water and removal of metal contaminants [5,6,7], it has also been shown that SO_4_ content of AMD waters and circumneutral acid mine waters can be reduced to an acceptable limit using fly ash [8,9].

The conversion of coal fly ash into high value zeolites is another way of minimizing the problems associated with fly ash disposal [5,10,11]. Recently, it has been shown that South African coal fly ashes belong to class F category [12,13], and provide a suitable feedstock for the synthesis of a pure phase zeolite Na-P1 [13,14].A recent study by Musyoka *et al.* [13] has shown that a pure phase Zeolite Na-P1 with high cation exchange capacity (4.11 meq/g) and surface area of 67 m^2^/g can be synthesized from South African coal fly ash, the product showed promise in the removal of toxic metals from industrial and/or municipal wastewaters. The authors reported optimum operating conditions of47 °C for 48 hours of ageing at a stirring speed of 800rpm using a magnetic stirrer followed by hydrothermal treatment at 140 °Cfor 48 hours under static condition [13].However the study was conducted on a small/laboratory scale. The commercial production of zeolite Na-P1 from waste fly ash will improve its potential application for treatment of wastewater, in addition to reducing the impact of fly ash disposal.

Successful commercialization of zeolite Na-P1 would require its production on a large scale, however an agitated vessel/reactor would be required [15]. Although the rheology of the slurry will play an important role for scale up production, agitation rate of 800 rpm using a magnetic stirrer [13] is impractical in an industrial sized reactor. The power requirement for an industrial sized impeller rotating at 800rpm may be exceedingly excessive to achieve the homogeneity of the viscous gel of reaction mixture as described by Casci [15]. Inadequate or ineffective mixing would result in an inhomogeneous reaction mixture, *i.e.*, reaction mixture may consist of pockets of gel having different compositions and consistency, each “pocket” behaves like a mini reactor and generates zeolite phases corresponding to the composition in that mini reactor. Agitation assists in the initial gel formation, reagent dissolution, maintaining a homogeneous gel and maintaining a uniform temperature across the reactor [15].

Shearing due to agitation is considered to be detrimental to the stability and purity of the zeolitic [16,17] product [13,14];this parameter appears to be significant during zeolite synthesis. In the study conducted by Marrot and his co-workers [16], it was shown that shearing seems to have a harmful effect on the stability and purity of the zeolitic product only during the crystallization (hydrothermal treatment) step of the synthesis process. Weitkamp and Puppe [17] reported that the effect of this physical parameter (agitation) can lead to a change of the attributes of the synthesis gel and also affect the outcome of the zeolite process. However, it is believed from the foregoing that during the ageing step shearing may be required in order to facilitate the dissolution of fly ash into the alkaline solution [13,18]. It should be noted that different impellers shear materials differently; therefore it is necessary to investigate the effect of impeller design and agitation during the ageing step of the zeolite synthesis process. The aim of this study was to investigate the effect of agitation rates and impeller designs during the ageing step on the synthesis of zeolite Na-P1 with a view to providing an understanding of how to attempt large scale production of a pure phase zeolite Na-P1 from South African coal fly ash.

## 2. Results and Discussion

### 2.1. Fly Ash Characterization

#### 2.1.1. X-ray Fluorescence

The elemental chemical composition of the South African fly ash used in this study is depicted in Table 1. The fly ashcan be classified as class F type (according to the ASTM standard C618) as reported previously [6,13]; the sum of SiO_2_, Al_2_O_3_ and Fe_2_O_3_ content is greater than 70%.This class of fly ash is formed from the burning of harder, older anthracite and bituminous coal [4]. Structurally, zeolites are made of tetrahedra of SiO_4_ and AlO_4_ joined together by sharing all oxygen atoms [19].This makesSiO_2_/Al_2_O_3_ ratio of the fly ash very important, as it will govern the Si/Al ratio of the final zeolite product. The average SiO_2_/Al_2_O_3_ ratio the fly ash used in this study was found to be 1.99 and it is appropriate for the synthesis of low Si-zeolites with high cation exchange capacity [20].This fly ash was also found to contain some potentially toxic trace elements such as As, Pb and Ba. Amongst the dominant trace elements present in fly ash, Sr, Ba, Ce, Zr and Th were found to be present in significantly higher concentration relative to the other traces in the ash. The elements not listed in the table were not analyzed for.

**Table 1:** Chemical composition of the South African fly ash used in this study.

Major oxides		Trace elements	
Oxides	(Mean mass %)	Elements	Concentration (ppm)
SiO_2_	55.66	As	49.10
Al_2_O_3_	27.95	Ba	708.93
Fe_2_O_3_	3.22	Ce	128.45
MnO	0.04	Co	43.87
MgO	1.91	Cu	46.88
CaO	4.38	Nb	58.10
Na_2_O	0.31	Ni	24.40
K_2_O	0.45	Pb	56.56
TiO_2_	1.13	Rb	29.29
P_2_O_5_	0.26	Sr	1011.45
SO_3_	0.03	V	113.49
Loss On Ignition	4.74	Y	84.78
Sum (%)	100.07	Zn	42.09
SiO_2_/Al_2_O_3_	1.99	Zr	442.60
-	-	Mo	11.28
-	-	Th	384.21

#### 2.1.2. X-ray Powder Diffraction

**Figure 1:**
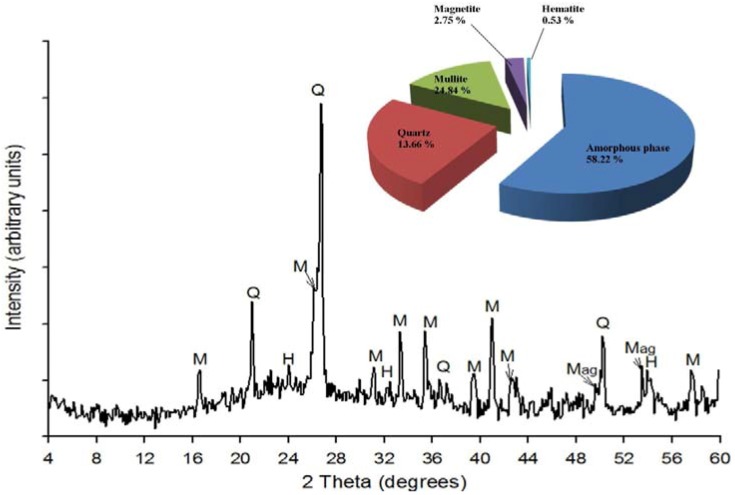
XRD results of the fly ash (Q-Quartz, M-Mullite, Mag-Magnetite and H-Hematite).

Qualitative and quantitative XRD analyses (Figure 1) of the raw fly ash used in this study shows that the major crystalline phases in the ash were quartz (SiO_2_), Mullite (3Al_2_O_3_.SiO_2_) with small amounts of magnetite and hematite. Quartz had the most intense peak at 26.85 degrees 2*θ* while the less intense peaks on the XRD patterns were identified as mullite, hematite and magnetite. The fly ash contained an amorphous glassy phase giving rise to a broad hump in the region between 18 and 32 degrees 2*θ* as indicated in the XRD spectra.

The overall quantitative XRD analysis confirmed that the fly ash used in this study was composed of phases that had been observed through qualitative XRD analysis (*i.e*., quartz, mullite, amorphous phase, magnetite and hematite). The quantification results in Figure 1 (see inset) show that the quantities of the mineral phases were; amorphous phase (58.22%), quartz (13.66%), mullite (24.84%), magnetite (2.75%) and hematite (0.53%).

#### 2.1.3. Morphology by SEM

**Figure 2:**
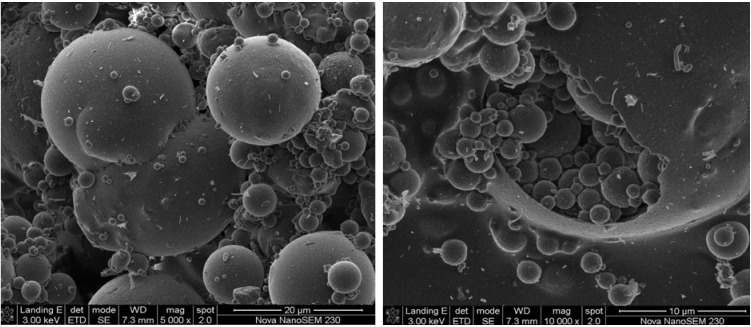
SEM images of fly ash morphology.

The SEM micrographs revealed that fly ash particles are predominantly spherical in shape with a relatively smooth surface texture and a wide particle size range (Figure 2). As stated in literature, the smoothness of the surface of the fly ash particles can be attributed to the fact that these particles are covered with the amorphous glass phase [21].In some cases smaller particles are attached to the surface of larger particles, serving as substrates. The spherical shape of fly ash particles is formed as a result of relatively sudden cooling during combustion processes [22]. The spherical particles are either solid or hollow. The hollow spherical particles are known as cenospheres and are believed to be formed by the expansion of CO_2_ and H_2_O gas, evolved from minerals within the burning coal [23,24]. Cenospheres may contain some smaller particles in their interiors (plerosheres).

### 2.2. Product Characterization

#### 2.2.1. Mineralogical Composition

Although Musyoka [13] reported the use of magnetic stirring at 800 rpm during the ageing step of the zeolite synthesis as part of the optimum conditions for the synthesis of a pure phase of zeolite Na-P1, it is clear that the use of a magnetic stirrer at 800 rpm may not be valid in a scale-up operation. However, it is believed that agitation during ageing using different impellers at lower speeds would increase the dissolution rate of SiO_2_ and Al_2_O_3_ from the fly ash into the solution as compared to a magnetic stirrer at a higher speed. These experiments were conducted in order to determine the optimum impeller design that can be used for agitation during ageing in a scaled-up process. The influence of this parameter was evaluated by determining the impact of the impeller design on the purity and the quality of the final zeolite Na-P1—the desired product.

**Figure 3:**
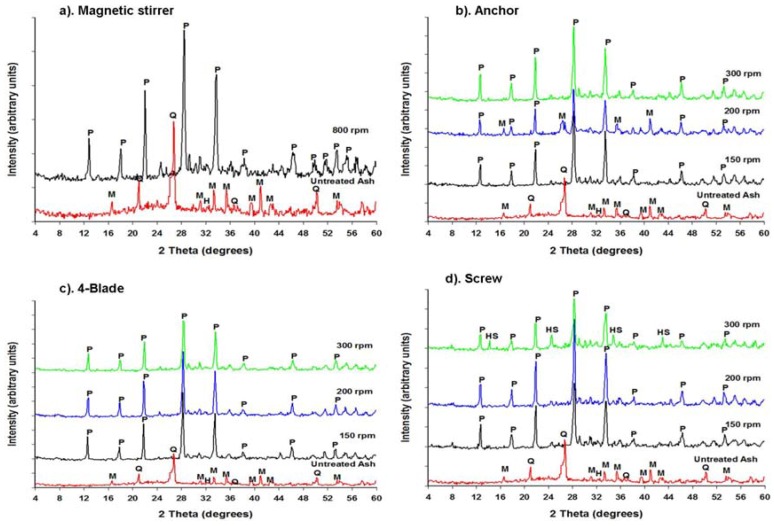
XRD spectra of the products synthesized using (**a**) Magnetic stirrer; (**b**) Anchor; (**c**) 4-Flat blade; and (**d**) Archimedes screw impeller at different stirring speeds during the ageing at 47 °C for 48 hours. (P = Zeolite Na-P1, Q = Quartz, M = Mullite, HS = Hydroxysodalite).

Musyoka *et al.* [13] indicated that a pure phase of zeolite Na-P1 (Na_6_Al_6_Si_10_O_32_12H_2_O) can be synthesized from coal fly ash using a magnetic stirrer during ageing to facilitate the dissolution of fly ash into the alkaline solution at 800 rpm. Their synthesis conditions were adopted in this study in order to demonstrate the reproducibility of previous data as well as creating a base case for the current study. The results obtained from this experiment provided a confirmation of Musyoka *et al.*’s findings. A pure phase of zeolite Na-P1 with a crystallinity of 93.0% was obtained as indicated in Figure 3a and Table 2 using a magnetic stirrer.

**Table 2:** Percentage crystallinity of zeolite Na-P1.

Impeller	Speed (rpm)	Crystallinity (%)	Impurities
4-Flat Blade	150	100	-
	200	100	-
	300	94.9	-
Screw	150	92.1	-
	200	94.7	-
	300	69.4	±30% hydroxysodalite
Anchor	150	91.0	-
	200	56.6	±40% mullite
	300	96.1	-
Magnetic Stirrer	800	93.0	-

In another set of experiments, various designs of impellers were used during the ageing step prior to static hydrothermal synthesis in order facilitate the dissolution of fly ash into the alkaline solution during ageing instead of a magnetic stirrer. The results obtained with the use of impellers during the ageing step were compared to those obtained with the use of a magnetic stirrer during ageing as shown in Figure 3. It was observed that all the three impellers investigated in this study could produce a pure phase of zeolite Na-P1 at lower agitation speeds.

It can be seen from the XRD patterns above that the fly ash phases were mostly completely converted into zeolite phases in all the experiments that were conducted. Firstly, a significant change was observed in the XRD patterns of the zeolitic products, namely the disappearance of the broad hump (between 18 and 32 degrees 2*θ*) as well as most quartz, hematite and mullite peaks signifying the dissolution of the amorphous phase and crystalline phases that were observed in the patterns of the raw fly ash. The disappearance of the various peaks signifies that the amorphous glassy phase as well as the more refractory quartz, mullite, hematite and magnetite phases were converted during the zeolite formation process. The major zeolite phase obtained after the activation of FA with NaOH solution and hydrothermal synthesis was zeolite Na-P1 (Na_6_Al_6_Si_10_O_32_.12H_2_O) (with the strongest peak at 28° = 2θ), with different yields, depending on the experimental conditions. Along with this zeolitic product, hydroxysodalite crystallized as a trace phase in the case of the screw type impeller at 300 rpm.

The XRD spectra of the products obtained with an anchor impeller (Figure 3b) showed that zeolite Na-P1 (Na_6_Al_6_Si_10_O_32_.12H_2_O) was the only zeolite phase synthesized. The XRD spectra of the products obtained with the abovementioned impeller at 150 and 300 rpm demonstrated a pure phase of zeolite Na-P1 with percentage crystallinity of 91 and 96.1% respectively. Unexpectedly, the characteristic XRD peaks of mullite and quartz still remained after 48 h of hydrothermal reaction at 200 rpm of stirring. This means that the product obtained with these conditions was a mixture of zeolite Na-P1 and fly ash since some of the quartz and mullite remained undissolved under the applied conditions. Mullite and quartz have been observed by several authors to show low or no reactivity in alkaline solutions [10,25]. Under this condition (anchor, 200 rpm), the crystallinity of zeolite Na-P1 was found to be 56.0%. In this case, it was concluded that the only source of Si and Al for zeolite crystallization was the amorphous glass phase since it has been found to be the most unstable and soluble phase in fly ash [10,11,21,25,26]. These experiments were repeated in order show the reproducibility of the results and similar results were obtained. The reason for the results obtained with the anchor at 200 rpm was unknown. However, it can be said that in order to obtain a pure high crystalline zeolite Na-P1 product with the anchor impeller, agitation must be performed at high speeds (>300 rpm).A 4-flat blade impeller was also investigated for the same activation conditions and agitation speeds.

In the set of experiments conducted with the use of a 4-flat blade impeller (Figure 3c), XRD showed that Na-P1 (Na_6_Al_6_Si_10_O_32_.12H_2_O) was the only phase that crystallized after the activation of fly ash with NaOH at all agitation speeds investigated (150, 200 and 300 rpm). The results obtained with the use of this impeller during ageing showed complete dissolution of fly ash, resulting in the crystallization of a pure phase of zeolite Na-P1 after the hydrothermal treatment step. The crystallinity of the products obtained after ageing under these conditions followed by static hydrothermal conditions is shown in Table 2.

Percent crystallinity of the samples was defined on the basis of the major (most intense) characteristic peaks of Zeolite Na-P1. The peaks used for these calculations were located and centered at 12.70, 17.90, 21.90, 28.30, 33.60 and 46.30 degrees 2*θ*. The major peaks were selected specifically because they are least affected by factors such as the degree of hydration of the samples and the type of cation compensation [16,27]. Percent crystallinity was calculated by using the Equation 1 [28].

(1) $$\%\;crystallinity\;=\;\frac{sum\;of\;area\;the\;under\;the\;XRD\;peaks\;of\;the\;product}{sum\;of\;area\;the\;under\;XRD\;peaks\;of\;the\;std\;sample}\;\times 100$$

Among all the synthesized zeolite Na-P1 samples, the one in which the sum of the diffraction intensities of these major peaks was highest was selected as the standard with 100% crystallinity and the relative crystallinity of the other samples was calculated according to that standard.

It can be seen that highly crystalline products with 100% crystallinity were obtained when ageing at lower speeds *i.e.*, 150 and 200 rpm using the 4-flate blade impeller. The crystallinity of zeolite Na-P1 decreased with an increase in agitation speed during ageing *i.e.*, from 100% at 200 rpm to 94.9% at 300 rpm when using the 4-flate blade impeller. The lower crystallinity of the product obtained under very vigorous 300 rpm high speed ageing with a high shear impeller seems to indicate that too high shear rate may not be advantageous. Based on these results, it can therefore be said that the use of a 4-flat blade impeller for agitation at moderate speeds during the ageing step leads to the crystallization of a pure phase zeolite Na-P1 after hydrothermal treatment step. This can be attributed to relatively high shearing that is produced by this type of impeller. The 4-flat blade impeller design is known to produce regions of high shearing around the blades (localized shearing) [29]. This facilitates the breaking of the Al-O and Si-O bonds in the fly ash. Hence, it resulted in a faster rate of dissolution of Al_2_O_3_ and SiO_2_ from the fly ash into the alkaline solution during ageing. Therefore, shearing is required during the ageing step in order to increase the amounts of Si^4+^ and Al^3+^ extracted from fly ash into the alkaline feedstock solution. Consequently, the use of a 4-blade impeller improved the dissolution of fly ash into the alkaline solution. Complete dissolution of fly ash is necessary in order to make available the feedstock required for the growth of zeolite crystals and this may lead to an increase in the yield of zeolites.

When comparing the results obtained with a 4-flat blade to those obtained with an anchor impeller (Figure 3b,c), it can be noticed that at low agitation speeds, *i.e.*, 150 and 200 rpm, a 4-blade impeller produced highly crystalline products with 100% crystallinity for both speeds compared to 91.0% at 150 rpm and 56.0% at 200 rpm with an anchor impeller. When comparing the 4-blade impeller to the other two impellers (Anchor and Archimedes screw) at all the speeds (150, 200 and 300 rpm), the 4-blade impeller appears to be the best impeller for agitation in the ageing step (Table 2), because of the highly crystalline products that resulted.

In the set of experiments conducted with the use of an Archimedes screw impeller (Figure 3d), XRD showed that Na-P1 was still the dominant phase formed, accompanied by traces of hydroxysodaliteat300 rpm but a pure phase of Na-P1 was obtained at 150 and 200 rpm. Since the product obtained at 300 rpm was a mixture of Na-P1 and hydroxysodalite, this was therefore regarded as an impurity. The formation of the hydroxysodalite phase may be due to several reasons. The successive transformation of zeolite Na-P1 into hydroxysodalite based on Ostwald’s rule of successive phase transformation may be one reason. This rule states that “the first phase to crystallize from a solution will be a hydrothermally least stable phase but with time, this phase will transform to more stable and denser phases” [30]. In addition, studies by Singer and Berkgaut [31] also pointed out that zeolite Na-P1 would be the first zeolite to form but found that it can be gradually be replaced by hydroxysodalite with increasing reaction time. Since the hydrothermal temperature and time were high (*i.e.*, 140 °C and 48 hours respectively), the extended synthesis time might be the reason for the formation of hydroxysodalite. Another reason may be, since the reaction mixture produces a viscous gel, if agitation is inadequate, an inhomogeneous reaction mixture may result with pockets of gel having different compositions and consistency. This leads to the crystallization of unwanted or different zeolitic materials [15]. The only difference noticed between the products obtained at 150 and 200 rpm with the screw impeller was the increase in the crystallinity of zeolite Na-P1 from 92.1% at 150 to 94.7% at 200 rpm. It can therefore be said that with a screw impeller, it is not necessary to stir at higher stirring speeds (>300 rpm) during ageing since a pure phase of zeolite Na-P1 with high crystallinity can be obtained at lower impeller speeds. The product yield of 0.98 ± 0.05 g zeolites/g fly ash obtained was an average for all the three impellers investigated at 200 rpm. Although a high yield was recorded, the process also generated significant amount of waste liquor, the re-useability has shown to be promising [32].

These investigations proved for the first time that different impeller designs and agitation during the ageing step can have a profound impact on the zeolitic product phase, purity and crystallinity. Therefore, it is not only the hydrothermal synthesis conditions and the molar regime but also the dissolution kinetics of the feedstock during ageing that influence the outcome of the zeolite synthesis process.

#### 2.2.2. Structural Analysis of the Synthesized Zeolites

**Figure 4:**
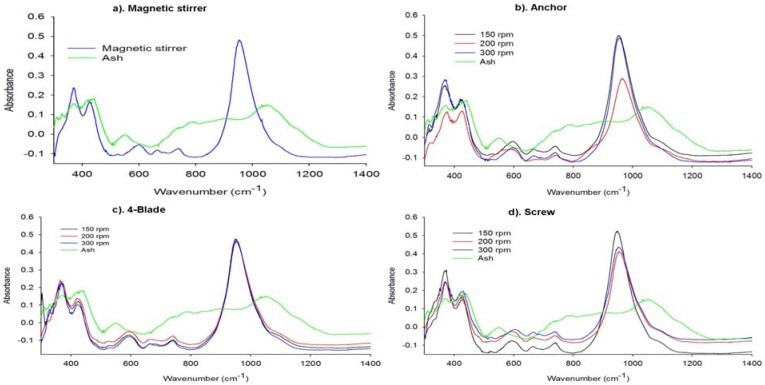
FTIR spectra of the zeolitic products synthesized using different impellers with variation of stirring speed.

The FTIR spectra bands were assigned in accordance with the generally accepted practice for the silicate and zeolite families of compounds [19].The FT-IR spectrum for FA (Figure 4) shows the three wide bands characteristic of aluminosilicates. The peak observed at 1053 cm^−1^ is associated with T-O (T = Si, Al) asymmetric stretching vibrations and may be attributed to the presence of quartz. The broadening of this peak may be due to the merging of FT-IR peaks corresponding to the other metal oxides present in FA. The broad bands appearing at 800 and 550 cm^−1^ can correspond to quartz and mullite, respectively, present in FA. The band at 420 cm^−1^ is associated with T-O bending vibrations [24].

In the FTIR spectra of the synthesized zeolite Na-P1 products, the characteristic vibration bands of fly ash disappeared, accompanied by the appearance of new bands that reveal the typical vibrations of zeolites. The IR vibrations common to all zeolites are the asymmetric stretching modes, which appear in the region 950–1250 cm^−1^ [19]. These bands have been reported to be sensitive to the Si/Al ratio of the product analyzed [19,33]. It can be seen that in all the IR spectrum of the synthesized zeolites (Figure 4a–d), the T-O band at 1053 cm^−1^ of the original fly ash became sharper and shifted perceptibly to lower frequencies. All these displacements are denoting that the vitreous component of the fly ash reacted with NaOH to form the zeolite structure. Since this study focuses on the effect of impeller design and agitation on the phase and purity of the zeolites synthesized, discussion on the effects of these parameters will be based on the changes that this band (T-O asymmetric stretching) undergoes depending on the experimental conditions. However, other bands common to all zeolites are discussed below. The next strongest bands are found in the 420–500 cm^−1^ region and are attributed the internal tetrahedron vibrations of the T-O bending mode [19]. These bands are also found in all zeolites. Bands in the region 300–400 cm^−1^ are assigned to the external linkages and are related to pore opening or the motion of the tetrahedral rings which form the pore opening of zeolites.

In the spectra shown in Figure 4a–d, it can be seen that the main zeolite band associated with the T-O asymmetric stretching vibrations (950–1250 cm^−1^) shifts perceptibly when compared to that in the original fly ash. This band shifted to the lower frequencies (around 850–1160 cm^−1^) with its center at approximately 950 cm^−1^. This can be attributed to the increase in the number of tetrahedrally positioned Al atoms in the zeolite product compared to the fly ash [19,33,34].

According to Criado *et al.* [35], the main zeolite band associated with the T-O asymmetric stretching vibrations provides information on the degree of crystallinity of a sample. This can be confirmed by looking at the IR spectra of the anchor impeller (Figure 4b). From these spectra, it can be seen that the intensity of this band in the product synthesized using 200 rpm during ageing was the lowest, signifying that the crystallinity of this product was low. This was also shown in the XRD results (Figure 3b, Table 2). In addition, the exact position of this band appears in slightly high wavenumbers (centered at 970 cm^−1^) than that of the products obtained at 150 and 300 rpm (centered at 950 cm^−1^). This basically means that the Si/Al ratio of this product (anchor, 200 rpm) may be slightly higher than that of the other products (150 and 300 rpm) as this band is sensitive to Si/Al ratio. Thus, this concurs with the results obtained from the XRD analysis of this product that fly ash did not completely dissolve as some of the Al was still incorporated in the mullite phase leading to increased Si/Al of the product.

The IR spectra of the products synthesized using a 4-flat blade impeller (Figure 4c) showed that bands of the T-O vibrations at different speeds overlap from 880 to 1060 cm^−1^(centered at 950 cm^−1^). This may be due to the Si/Al ratio and the concentration of these zeolite products being the same. Based on these results, it can be said that under these conditions (4-blade-150, 200 and 300 rpm) fly ash was well dissolved during ageing liberating the Si^4+^ and Al^3+^ from the solid phase (fly ash) into the solution (NaOH), leading to similar Si/Al of the zeolite products. These results concur with the results of the XRD data discussed previously. It can also be seen that with the screw impeller (Figure 4d), there was also complete conversion of fly ash. The T-O asymmetric stretching vibrational bands of all the zeolites products obtained with the screw impeller lie in the region of 850–1160 cm^−1^ wavenumbers with its center at approximately 950 cm^−1^. Thus, these products possess similar Si/Al ratio. Based on these observations, it can be said that FTIR is a good complementary technique for XRD, confirming the crystallinity of the zeolite product.

#### 2.2.3. Elemental Composition of the Synthesis Product

The XRF analyses were performed for most crystalline zeolite Na-P1 product as identified by XRD *i.e.*, the product obtained with 4-flat blade impeller at 200 rpm. The product used for this analysis were synthesized in triplicate under the abovementioned conditions, these were analyzed for the major oxides and elemental composition (Table 3). The average SiO_2_/Al_2_O_3_ ratio of these products was found to be 1.57±0.01 which was lower than that of the original feedstock fly ash shown in Table 1. This suggests that the unreacted SiO_2_ and Al_2_O_3_ might have been lost with the waste supernatant during the recovery stage; however the result shows that the trace elements were mostly retained with the zeolitic products. The Na_2_O content in the zeolites products was found to be higher than that observed in the fly ash. The increase in the content of this oxide can be attributed to the fact that the fly ash was activated by a solution of NaOH.

**Table 3:** Chemical composition (major oxides) of the zeolitic products (P1, P2, P3-Product 1, 2 and 3).

Major oxides (Mass %)			Trace elements (ppm)		
Oxides	Average*	Standard deviation*	Element	Average *	Standard deviation*
SiO_2_	42.60	1.90	As	47.15	2.25
TiO_2_	1.51	0.05	Ba	679.13	25.55
Al_2_O_3_	27.10	1.35	Ce	137.34	9.35
Fe_2_O_3_	4.53	0.17	Co	42.11	2.14
MnO	0.05	0.00	Cu	46.12	1.40
MgO	1.33	0.04	Nb	57.15	1.30
CaO	4.05	0.08	Ni	24.05	2.50
Na_2_O	10.45	0.70	Pb	51.35	3.22
K_2_O	0.26	0.05	Rb	32.23	3.85
P_2_O_5_	0.06	0.00	Sr	981.44	36.00
Cr_2_O_3_	0.03	0.00	V	111.35	2.65
NiO	0.01	0.00	Y	81.12	3.04
V_2_O_5_	0.01	0.00	Zn	39.00	3.00
ZrO_2_	0.05	0.00	Zr	420.55	14.5
CuO	*<0.01*	*<0.01*	*Mo*	*10.25*	*0.80*
LOI	7.62	4.19	Th	350.05	18.25
TOTAL	99.66	0.29	-	-	-
**SiO_2_/Al_2_O_3_**	1.57	0.01	-	-	-

* means standard deviation of triplicate values.

#### 2.2.4. Morphological Characterization by SEM

**Figure 5:**
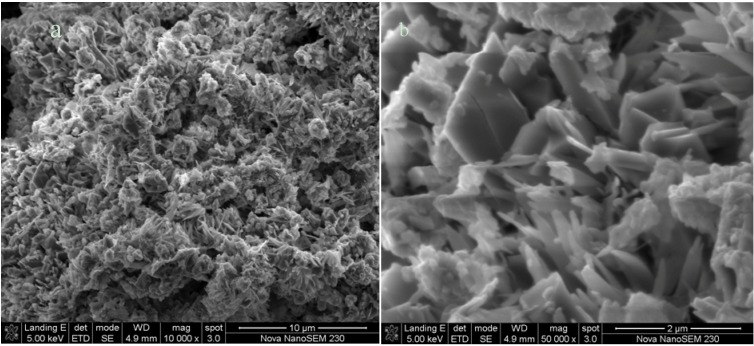
SEM images of zeolite Na-P1 synthesized with the 4-flat blade impeller at 200 rpm, both (**a**) and (**b**) show zeolite Na-P1 at different magnification.

Scanning Electron Microscopy analyses were done on only the most crystalline zeolite Na-P1 product (Figure 5). The zeolite product obtained with the use of a 4-flat blade impeller at 200 rpm was identified as the most crystalline zeolite product (100% crystallinity) based on the XRD and FTIR analysis. The N_2_-BET analysis of surface area and pore volume also agrees with the value reported by Musyoka *et al.* [13], values of 67 m^2^/g and 3.4 × 10^−3^ cm^3^/g were obtained respectively. The SEM micrographs of the surface of the synthesized zeolites revealed a clear transformation of the spherical particles characteristic of the fly ash (Figure 2) into needle-like crystalline structures. These needle-like structures were identified from XRD analysis as zeolite Na-P1. According to the literature, this morphology has been shown to be a typical morphology for zeolite Na-P1 [36].

## 3. Experimental Section

### 3.1. Materials

Samples of pulverized fly ash used in this study were collected froma South African power station. The samples were stored to maintain their initial overall phase compositions as described in a previous study [18].The sodium hydroxide pellets (Merck) were used as the source of sodium for zeolite synthesis.

### 3.2. Preparation of Zeolites

**Figure 6:**
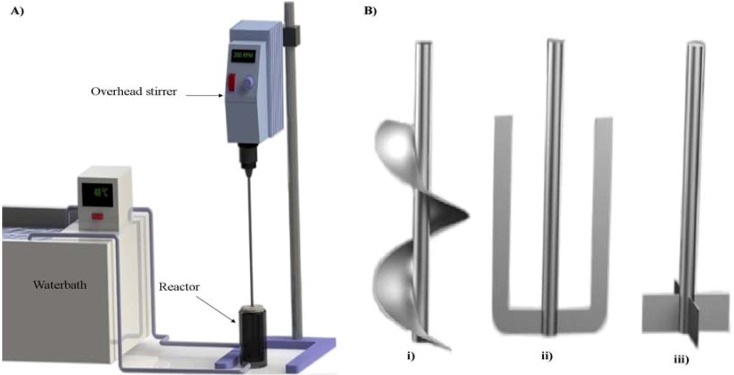
(**A**) Experimental set-up of the ageing process; (**B**) Three types of impellers used in this study *i.e.*, (i) Archimedes Screw; (ii) Anchor; (iii) 4-Flat blade impeller.

A two step synthesis procedure which consisted of ageing and hydrothermal treatment adopted from Musyoka *et al.* [13] was followed in this study. The ageing step (Figure 6a) was conducted by mixing fly ash with a 5M NaOH solution in a 150 mL glass reactors at a fly ash/solution ratio of 0.2 g/mL *i.e.*, 20 g of fly ash in 100 mL of NaOH solution [13].Three types of impellers (Figure 6b) were designed for the identical glass reactors according to the specification described by Casci [15], each was used to agitate the slurry in an identical fashion and at different stirring speeds of 150, 200 and 300 rpm. However, it must be noted that our choices of impeller design were such that they range from high shearing impeller (4-blade)to a low shearing (screw blade impeller) while still maintaining complete mixing. The ageing temperature and time were kept at 47 °C and 48hours as reported [13]. After the ageing period, 150 mL ultra pure water was added to the synthesis gel while stirring. The resulting homogeneous mixture was then transferred into 23 mL Parr bombs. Crystallization of the feedstock was achieved by placing the mixture in sealed Parr bombs under static, unstirred conditions in a hot air oven for a period of 48 hours at a temperature of 140 °C. Each of the experiment gave a space-time-yield (STY) of 15 ± 0.4kg d^−1^ m^−3^ as determined using Equation 2.The zeolitic products were recovered by filtration followed by thorough washing with de-ionized water until the pH of 9–10 of the filtrate was achieved. The details of the hydrothermal treatment in using the Parr bombs have been described extensively elsewhere [18]. The zeolites products were then dried in an oven overnight at 90 °C and ground into powder for further analysis.

(2) $$STY\;=\;\frac{m}{tV}$$

where m = mass of product; t is the total synthesis time (days) and V is the total reaction volume (m^3^).

### 3.3. Characterization Techniques

The mineral phases in raw fly ash and synthesized zeolitic products were identified using Philips X-ray diffractometer operating with Cu-Kα radiation (40 kV and 40 mA). Data collection was carried out in the 2*θ* range 4–60°, with a step size of 0.1°. The phase identification was performed by searching and matching obtained spectra with the powder diffraction file database with the help of JCPDS (Joint committee of powder diffraction standards) files for inorganic compounds. Chemical composition of the fresh ash was carried out on a Philips PW 1480 X-ray spectrometer. Major elements were analyzed on a fused glass bead at 40 kV and 50 mA tube operating conditions and trace elements were analyzed on a powder briquette at 50 kV and 40 mA tube operating conditions. The morphology of the fly ash and zeolites prepared in this investigation was examined using the Cambridge S200 SEM which is equipped with an energy dispersive X-ray analyzer. Attenuated total reflectance (ATR) FTIR analysis was carried in order to gain an insight about the molecular structure of the product. A Perkin Elmer spectrum 100 FT-IR spectrometer was used to carry out this analysis and the samples were scanned in the range between 250 and 4000 cm^−1^.

## 4. Conclusions

A pure phase of zeolite Na-P1 was successfully synthesized with the use of different impellers at different speeds during the ageing step. In some cases, zeolite formation was assessed to be fairly unsuccessful, taking into account that the synthesis products were either of low crystallinity or contained a small percentage of hydroxysodalite or mullite. Based on the experimental results, it was observed that the 4-flat blade impeller was the optimum design of impeller that can be used for agitation during the ageing step of the zeolite synthesis in a scaled-up process. This study showed for the first time that different impeller designs and agitation during the ageing step prior hydrothermal synthesis can have a profound impact on the zeolitic product quality. Therefore, in addition to the hydrothermal synthesis conditions and the molar regime, the dissolution of fly ash during the ageing step also influences the outcome of the zeolite synthesis process.
